# Mechanistic insight into female predominance in Alzheimer’s disease based on aberrant protein S-nitrosylation of C3

**DOI:** 10.1126/sciadv.ade0764

**Published:** 2022-12-14

**Authors:** Hongmei Yang, Chang-ki Oh, Haitham Amal, John S. Wishnok, Sarah Lewis, Emily Schahrer, Dorit Trudler, Tomohiro Nakamura, Steven R. Tannenbaum, Stuart A. Lipton

**Affiliations:** ^1^ Departments of Biological Engineering and Chemistry, and Center for Environmental Health Science, Massachusetts Institute of Technology, Cambridge, MA 02139, USA.; ^2^ Northeast Asia Institute of Chinese Medicine, Changchun University of Chinese Medicine, Changchun 130021, China.; ^3^ Department of Molecular Medicine and Neurodegeneration New Medicines Center, The Scripps Research Institute, La Jolla, CA 92037, USA.; ^4^ Institute for Drug Research, School of Pharmacy, Faculty of Medicine, The Hebrew University of Jerusalem, Jerusalem, Israel.; ^5^ Department of Neurosciences, School of Medicine, University of California, San Diego, La Jolla CA 92093, USA.

## Abstract

Protein S-nitros(yl)ation (SNO) is a posttranslational modification involved in diverse processes in health and disease and can contribute to synaptic damage in Alzheimer’s disease (AD). To identify SNO proteins in AD brains, we used triaryl phosphine (*SNO*TRAP) combined with mass spectrometry (MS). We detected 1449 SNO proteins with 2809 SNO sites, representing a wide range of S-nitrosylated proteins in 40 postmortem AD and non-AD human brains from patients of both sexes. Integrative protein ranking revealed the top 10 increased SNO proteins, including complement component 3 (C3), p62 (SQSTM1), and phospholipase D3. Increased levels of S-nitrosylated C3 were present in female over male AD brains. Mechanistically, we show that formation of SNO-C3 is dependent on falling β-estradiol levels, leading to increased synaptic phagocytosis and thus synapse loss and consequent cognitive decline. Collectively, we demonstrate robust alterations in the S-nitrosoproteome that contribute to AD pathogenesis in a sex-dependent manner.

## Teaser

Aberrant protein S-nitrosylation of complement C3 contributes to the increased prevalence of Alzheimer’s disease in females.

## INTRODUCTION

Neurodegenerative diseases, characterized by a progressive loss of brain function, result primarily from synaptic loss and neuronal cell death in the central nervous system (*1*, *2*). Alzheimer’s disease (AD)—characterized by the accumulation of misfolded amyloid-β (Aβ) peptide and neurofibrillary hyperphosphorylated tau tangles in the brain—is arguably the most common neurodegenerative disorder leading to dementia (*3*). The etiology and pathogenesis of AD are incompletely understood, and effective, disease-modifying drug treatments are lacking (*4*).

Prior work has shown that genetic, environmental, and age-related factors, along with alterations in energy metabolism, autophagy, and synaptic function, all contribute to the pathogenesis of neurodegenerative diseases such as AD (*5*–*8*). Oxidative/nitrosative redox stress, immune system alterations, and neuroinflammation may be proximate mechanisms (*9*–*11*). Considerable evidence suggests that posttranslational modifications (PTMs) of proteins regulate various signaling pathways; for example, phosphorylation and ubiquitination may be critical for disease progression in many disorders (*12*–*14*). To further elucidate the mechanism(s) underlying the critical neurodegenerative processes in AD, assessment of molecular networks using a proteomics strategy may be fruitful (*6*, *15*, *16*), and an untargeted proteomics approach offers identification and quantification of many proteins, which can then identify biological processes characteristic of a disease.

Nitric oxide (NO·) — the free radical product of NO synthases (NOSs)—regulates diverse physiological processes, including vascular homeostasis and various host defense systems (*17*–*19*). NO-related species may act via protein S-nitrosylation (SNO), a reversible chemical reaction in which the NO group binds to a cysteine thiol/thiolate group (*20*), either through free radical recombination, transition metal-intermediate, or transnitrosylation from another protein or peptide (*21*). SNO can influence protein activity, localization, conformation, or interactions with other proteins; aberrant protein SNO may play a key role in the pathogenesis of various neurodegenerative diseases (*22*–*27*). For example, SNO is key to posttranscriptional modification of the core autophagy machinery (*27*). In many cases, SNO is a relatively labile modification and can be reversed in the presence of various metal ions, ascorbic acid, or glutathione; in other cases, denitrosylating enzymes may be involved (*28*–*30*).

Accurate quantification of SNO proteins is technically challenging. One enrichment approach for SNO analysis involves indirect detection via the biotin-switch technique (*31*–*33*), which requires blocking of free thiols, followed by selective reduction with ascorbate of SNO-Cys residues that are then biotinylated. A limitation of this approach is that incomplete blocking of free Cys thiols and nonselective reduction of protein SNO can lead to false positives (*34*). Accordingly, various chemical proteomics strategies have been developed for improved analysis of SNO proteins, using methods such as organomercury-based enrichment of S-nitrosothiols followed by mass spectrometry (MS) analysis (*35*), bio-orthogonal cleavable-linker–based enrichment and switch technique (*36*), modified biotin-switch assay using resin-assisted capture (SNO-RAC) (*37*), and, as used here, *SNO*TRAP (SNO trapping with a triaryl phosphine probe) (*38*, *39*). Proteomics also plays an increasing role in the search for biomarkers of disease (*40*), and thus, improved techniques are critical for this line of research.

In the present study, we applied an innovative approach based on analysis of the tryptic SNO peptides derived from SNO proteins, using a modified *SNO*TRAP method combined with nano–liquid chromatography (LC) Orbitrap MS. This technique provides the advantage over other approaches of direct reaction of probe with S-nitrosothiol and yielded 1449 SNO proteins and 2809 SNO sites in postmortem human brain samples from AD and non-AD patients. Comparison of male AD and female AD brain proteomes revealed both similarities and differences, reminiscent of our previous findings in wild-type (WT) mice (*41*). Detailed bioinformatic analysis revealed altered metabolic pathways and other biological processes. Increased exposure to endogenous NO in AD brains compared to age- and sex-matched control brains had been previously demonstrated by assessing expression of NOS isoforms (*42*). Here, we found that several key proteins and pathways known to be involved in AD were associated with aberrant protein SNO, particularly affecting the complement pathway, for example.

Although tremendous strides have been made in AD research over the past decade, additional consideration of sex differences will be important to explain the increased incidence of disease in females (*43*). Recently, sex differences have been observed at the transcriptional level (*44*) and terms of genetic and hormonal factors (*45*). Along these lines, we found sex to be a significant covariate in our study. Differences between sexes were reflected in potential SNO proteins in AD brain of females compared to males. These differences may reflect the fact that the disease predominates in women almost twofold over men (*46*).

To our knowledge, this is the first investigation comparing changes in NO-modified protein levels in the brains of male and female humans with AD. These results should serve as a resource providing a large library of SNO-cysteine sites to other scientists to encourage additional research, facilitate better understanding of the etiology, and ultimately prevent and treat AD.

## RESULTS

### Site-specific identification and label-free quantification of SNO proteins reveal differences between AD and non-AD groups

**Fig. 1.**
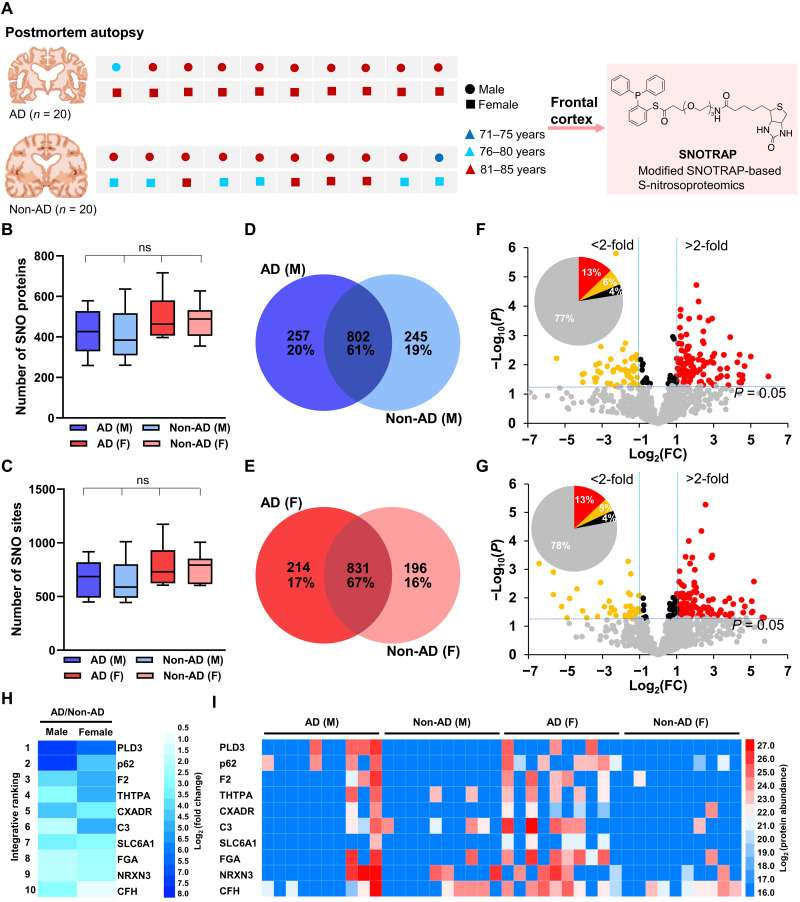
Characterization of SNO-proteins in male (M) AD and female (F) AD versus non-AD brains by SNO proteomics. (**A**) Age- and sex-matched AD and non-AD control brains used in this study. (**B** and **C**) The number of SNO proteins (B) and SNO sites (C) in each human brain. Box-and-whiskers plots show the median, interquartile range, and minimum and maximum of SNO proteins and SNO peptides found per each group. Statistics were performed with analysis of variance (ANOVA) and Tukey’s post hoc test. *n* = 10 brains per group. ns, not significant. (**D** and **E**) Venn diagrams representing the number of SNO proteins identified in male AD and non-AD individuals (D) and in female AD and non-AD individuals (E). (**F** and **G**) Volcano plots of fold change (FC; log_2_) versus *P* value (−log_10_) of signal intensity for the 802 SNO proteins identified to be in common between male AD and non-AD samples (F) and from the 831 SNO proteins identified to be in common between female AD and non-AD samples (G). Pie chart shows percentage of the proteins. (**H**) Rank list of the top 10 up-regulated SNO proteins in male AD and female AD brains compared to respective non-AD brains. (**I**) Heatmap of the top 10 differentially up-regulated SNO proteins shown for each brain. Each protein is represented by a colored box reflecting log_2_ (protein abundance).

We performed a nanoLC–tandem MS (MS/MS)–based semiquantitative proteomics analysis using a modified *SNO*TRAP approach to find key SNO proteins that may contribute to the pathogenesis of AD. *SNO*TRAP selectively converts SNO to a stable disulfide-iminophosphorane in the SNO protein, followed by trypsin digestion. Then, the tryptic peptides are enriched on streptavidin agarose beads. The bound peptides are eluted, alkylated, and desalted. Although nano-LC is a common tool for separating peptides/proteins before MS, Orbitrap MS has improved resolution and sensitivity, which makes it an attractive technology for a wide range of proteomics analyses such as that described here. This procedure was carried out on flash-frozen samples of frontal cortex from 40 human brains with relatively short postmortem times (Fig. 1A and tables S1 and S2). This region was chosen because neuropathological examination revealed that it was affected by AD in these brains. In total, four different groups of brains were analyzed: male AD, male non-AD, female AD, and female non-AD. For a list of proteins identified in the four different groups, see table S3. Analysis of technical and biological replicates was necessary to ensure the accuracy and biological significance of the proteomic datasets. We did not pool samples for MS analysis to monitor possible individual variations. Proteins were identified in each biological replicate and then compiled into a single list.

We identified 1450 SNO proteins and 2809 SNO sites from all of the human brain tissues; only 33 proteins could not be identified, as expected in the analysis of MS spectra, corresponding to a specificity of 98%. This information provides a larger database than previously identified for SNO proteins and SNO sites (table S4). For example, our data greatly surpassed our previously reported 135 SNO proteins in the mouse brain using the *SNO*TRAP strategy (*39*). A case-by-case review (table S5) shows that there was no significant difference in the number of SNO proteins (Fig. 1B) or SNO sites (Fig. 1C) between the disease and non-AD groups. On average, we identified 464 SNO proteins and 729 SNO sites per AD brain and 447 SNO proteins and 708 SNO sites per non-AD brain. However, the disease process did not just result in the addition of SNO sites but rather a restructuring of the SNO proteome, as elaborated below.

Overall, a total of 1304 and 1241 SNO proteins were identified in the male and female groups, including 257 and 214 SNO proteins found exclusively in male AD and female AD brains, respectively, with extensive but not perfect overlap between the male and female groups (Fig. 1, D and E, fig. S1, and table S6). Approximately 60% of the SNO proteins were in common between AD and non-AD groups, meaning that 40% of these proteins were not found to be in common.

When a SNO protein appeared in both AD and non-AD brains, label-free quantification based on peak intensity using Spectrum Mill software was carried out, and analyzed separately from the SNO proteins found exclusively in AD versus non-AD control brains mentioned above, with quality control measures to determine whether there was a statistical difference in the levels. Volcano plots (Fig. 1, F and G) showing the scatter of fold change (FC) versus *P* value for each protein were used to interpret the results of this quantitative analysis. Red and orange circles represent significant (*P* < 0.05) ≥2-fold up-regulated and down-regulated proteins, respectively. Black circles represent significant (*P* < 0.05) changes but somewhat less than twofold. The blue horizontal dotted line represents *P* = 0.05, and the gray circles below this line are thus not significant. The SNO proteins represented in the extreme upper left and right corners of the volcano plot, which manifest the smallest *P* values and largest absolute FC, show the largest and statistically most significant changes in expression. The percentage of proteins in each section was very similar among the datasets. The four lists of differentially expressed SNO proteins (SNO proteins up-regulated and down-regulated in male or female AD brains) that reached statistical significance together with their FC and *P* values are presented in table S7. Consideration of both the SNO proteins exclusively found in AD brains (and not in non-AD brains) and those that were found to be statistically increased in AD brains over non-AD brains yielded an integrative protein ranking (table S8) that showed phospholipase D3 (PLD3), p62 sequestosome 1(SQSTM1), and complement component 3 (C3) were among the top S-nitrosylated proteins on the list (Fig. 1H), potentially suggesting previously unknown avenues of pathogenesis for AD. The detailed abundance of the top 10 proteins in each brain is depicted in Fig. 1I. The analysis also revealed that several of the SNO proteins were differentially expressed in female versus male AD brains.

In addition, we analyzed our smaller dataset of non-AD control brains that were completely “normal” (displaying no neuropathology indicating any form of dementia) versus AD brains (as listed in table S1). Although the *P* value was greater than 0.05 in most cases due to the limited number of normal brains available to us for this study (*n* = 6, four male and two female), we were able to detect a number of significantly up-regulated proteins with a *P* value of <0.05. In particular, PLD3, C3, and p62 were still present in the top 10 SNO proteins in the analysis of AD brains versus normal brains (table S9).

### Enrichment of SNO proteins associated with gene ontology processes and pathways known to be affected in AD

In an effort to establish functional connections between identified SNO proteins and test whether the enriched processes are related to the pathogenesis of AD, we applied stringent bioinformatic criteria using gene ontology (GO) and pathway analysis on the differentially expressed SNO proteins using MetaCore software (final lists in table S10). We identified GO processes and pathways with statistically significant enrichment of S-nitrosylated proteins within the proteome and then focused on the subset of these processes and pathways that were differentially affected in AD versus non-AD control brains (fig. S2, detailed lists in table S11). Some of these pathways were found in common between male and female AD brains, while some of the pathways were unique to either male or female AD brains. For example, a top-ranked GO pathway for both male and female AD brains (fig. S2, A and B) was “Immune response-Classical complement pathway,” in line with the role of neuroinflammation in AD. Another pathway known to be of interest in AD brains is “Oxidative stress_ Activation of antioxidant defense system.” In this case, *SNO*TRAP identified 5 of 60 proteins annotated in this pathway in male, but not female, AD brains (fig. S2A); these S-nitrosylated proteins included catalase (CAT), p62, peroxiredoxin 3 (PRDX3), PRDX1, and liver kinase B1 (LKB1), with a *P* value of 4.4 × 10^−3^ and false discovery rate (FDR) of 0.09. SNO proteins associated with this oxidative stress pathway were not significantly enriched in non-AD male brains, emphasizing the association of these SNO proteins specifically with AD.

### SNO protein reactome pathway analysis reveals enrichment of complement cascade in AD human frontal cortex

In addition to functional clustering analyses, we used String analysis to explore possible interactions among the top up-regulated and down-regulated SNO proteins. Network clusters for each of the four groups is presented in table S12. In total, 244, 157, 177, and 129 SNO proteins passed our cutoff criteria and were classified as interactors in male AD, male non-AD, female AD, and female non-AD brains, respectively.

Further analysis of human AD brains by String interactome analysis showed networks of SNO proteins that are positively correlated with several processes thought to be involved in AD (fig. S3, A and B). For example, pathway enrichment analysis in the male-AD group revealed that pathways involving mitophagy mediated by PTEN induced kinase 1 (PINK1)/parkin [encompassing autophagy protein 5 (ATG5), p62, and voltage dependent anion channel 1 (VDAC1)], metabolism regulated by the tricarboxylic acid cycle and mitochondrial respiratory chain, immune responses of the complement cascade [C3, complement factor H (CFH), prothrombin (F2), etc.], and cellular responses to stress [calcium/calmodulin dependent protein kinase II alpha (CAMK2A), CAT, PRDX2, etc.] were overrepresented in the up-regulated SNO proteins (fig. S3A). Notably, regulatory proteins and pathways clustered in the aged male non-AD group also included the innate immune system and metabolism-related pathways (table S12), indicating that these pathways, while found to be affected in male AD brains over non-AD brains (fig. S3A), could reflect the known AD risk factor of aging. Analysis of the female AD dataset compared to female non-AD revealed pathways associated with cell-surface interactions at the vascular wall, amyloid fiber formation, the Rho guanosine triphosphatase (GTPase) cycle, and platelet activation, signaling, and aggregation (fig. S3B).

### Sex-specific protein SNO in human AD brains

**Fig. 2.**
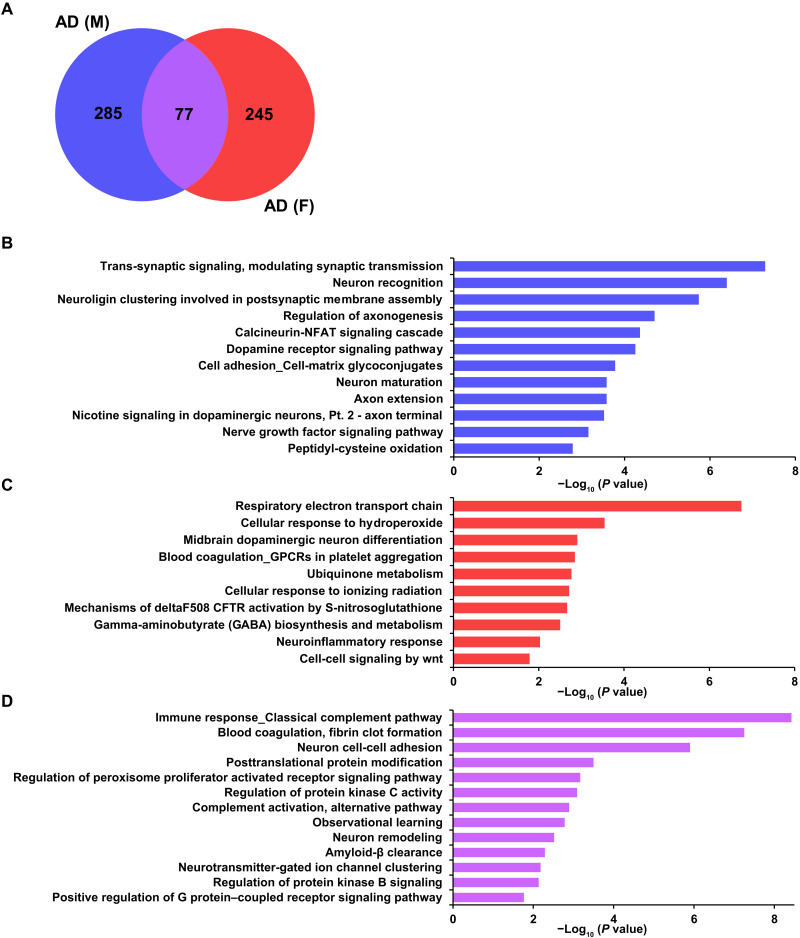
Differences in the S-nitrosoproteome between male and female AD human brains from a systems biology analysis. (**A**) Venn diagram represents SNO proteins that were quantitatively up-regulated in the frontal cortex of male and female AD brains. (**B** and **C**) GO and pathway analysis conducted on SNO proteins exclusive to male AD cortex (B) and female AD cortex (C). (**D**) GO and pathway analysis conducted on SNO proteins found to be in common between male and female AD frontal cortex.

To interrogate further the hypothesis that sex affects the SNO proteome, Venn diagrams were generated to compare SNO proteins that were quantitatively up-regulated to a statistically significant degree in male AD and female AD brains compared to their respective non-AD control brains (Fig. 2A). In the frontal cortex, we identified a total of 285 proteins that were S-nitrosylated exclusively in male AD brains, 245 proteins S-nitrosylated exclusively in female AD brains, and 77 SNO proteins found to be in common (see comparison lists in table S10). With the goal of evaluating differences in molecular processes between male AD and female AD brains, pathway and GO analysis of the three datasets of SNO proteins in Fig. 2A were carried out. As expected, analysis of the male and female AD brain samples revealed significant enrichment of GO terms and pathways that have been strongly associated with AD, such as the “Calcineurin-NFAT signaling cascade” in male AD (Fig. 2B) and “Respiratory electron transport chain” in female AD (*47*, *48*) (Fig. 2C). Other differential responses in male and female AD brains are presented in Fig. 2 (B and C). Pathways or GO processes found in common among male and female AD brains, as alluded to above, include “Immune response_Classical complement pathway” and “Complement activation, alternative pathway” (Fig. 2D). Collectively, these biological process terms and pathway analyses provide insight into potential SNO-mediated dynamic regulation of proteins during the pathogenesis of AD. For a complete list of the GO processes and pathways enriched for SNO proteins in all three datasets (representing AD brains of males only, females only, or both sexes), see table S13.

**Fig. 3.**
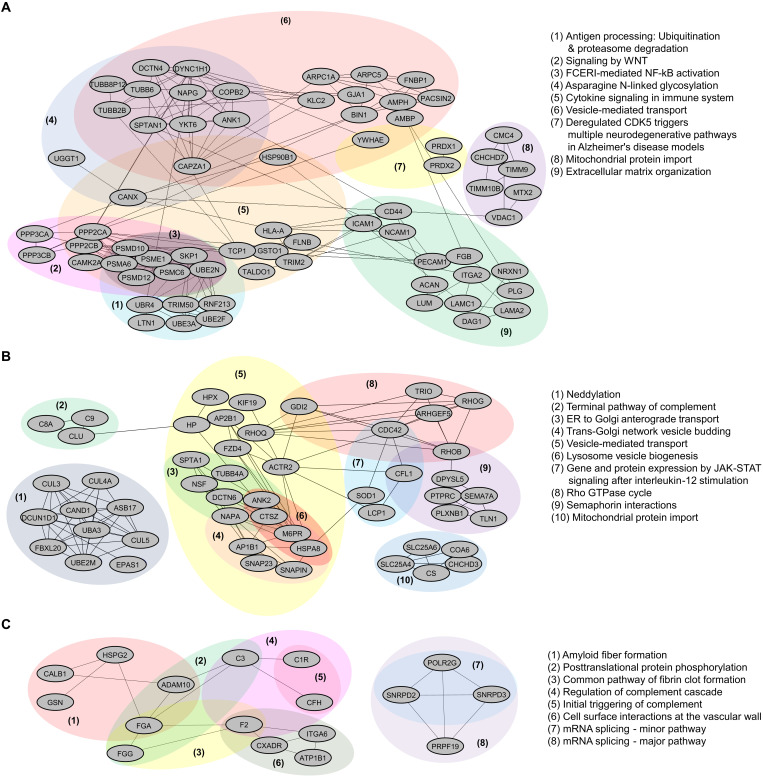
Cluster analysis of SNO proteins from male and female AD cortex (from Fig. 2). (**A** and **B**) Interaction network of SNO proteins exclusively found in male AD cortex (A) and female AD cortex (B). (**C**) Interaction network analysis conducted on the set of SNO proteins found to be in common between male and female AD cortex. Regulated functional protein clusters or complexes are indicated by shadows.

To further investigate possible sex-specific changes in human AD brains, these three datasets (as depicted in Fig. 2A) were also subjected to interactome analysis. We found 225 reactome (RCTM) pathways enriched for SNO proteins found in male AD brains and 37 RCTM pathways in female AD brains (table S14). Network analysis of the SNO proteins in the male and female AD datasets therefore clearly showed distinct multifunctional protein networks (Fig. 3, A and B). In the male SNO protein dataset, proteins were found to be involved in networks designated as “FCERI-mediated NF-κB activation network,” “Deregulated CDK5 triggers multiple neurodegenerative pathways in Alzheimer’s disease models,” and “WNT signaling” (Fig. 3A). In contrast, in the female AD dataset of SNO proteins, networks were enriched for “terminal pathway of complement,” “Rho GTPase cycle,” and “Semaphorin interactions,” among others (Fig. 3B). This comparison of networks involving SNO proteins suggests that molecular diversity at the level of sex exists in AD pathology. Although these analyses support an underlying sex difference in molecular processes linked to AD, further analyses of larger sample sizes and additional experimental follow-up studies will be needed to fully understand the pathological differences between sexes. Intriguingly, RCTM pathway analysis of SNO proteins revealed that the same pathway of “mitochondrial protein import” was affected in both male and female AD brains despite the fact that this link was based on different SNO proteins that were not present in the common dataset (viz. Fig. 3, A and B). This finding indicates that SNO can affect similar networks of proteins in the two sexes even if the individual proteins that are S-nitrosylated are distinct—this fact tells us that network analysis is crucial to understanding the overall effect of protein SNO in a disease process.

In addition, upon analyzing the 77 SNO proteins found to be in common between male and female AD brains, similar to our prior GO and pathway analyses, RCTM analysis implicated processes affecting amyloid fiber formation and the complement cascade (Fig. 3C). Four proteins were found to be shared interaction partners in the regulation of the complement cascade: C3, F2, C1R, and CFH. This result strongly implicates a role for protein SNO in regulating complement in AD brains (*49*). Complement activation is known to mediate inflammatory pathways in response to cell stress and thus contribute to neuronal dysfunction (*50*), so potential biochemical regulation of complement activity in the AD brain could prove important to pathogenesis.

### Individual highly enriched S-nitrosylated proteins in AD brains

**Fig. 4.**
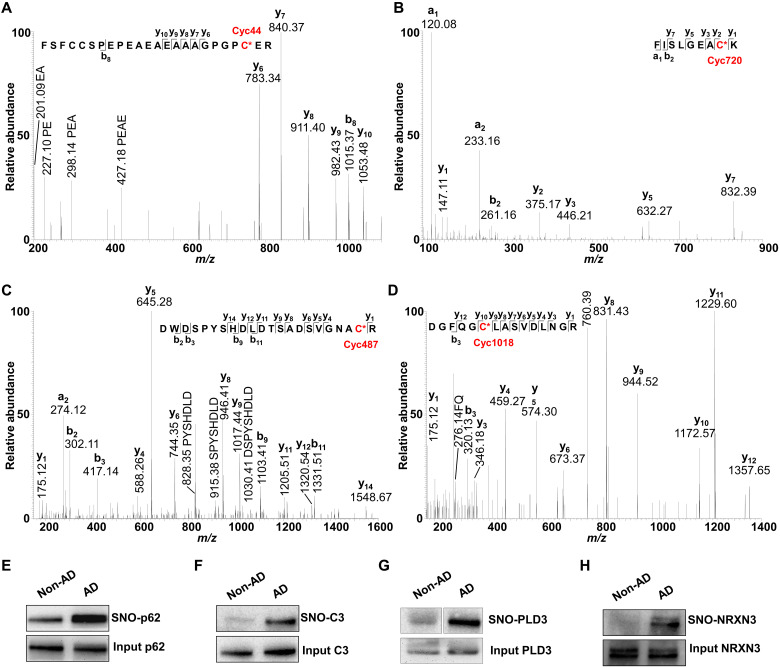
Representative tandem mass spectra of peptides displaying SNO of p62 (Cys^44^), C3 (Cys^720^), PLD3 (Cys^487^), and NRXN3 (Cys^1018^). (**A** to **D**) Spectra for p62 and C3, PLD3, and NRXN3, respectively. a-, b-, and y-type product ions are indicated. (**E** to **H**) Representative immunoblots of S-nitrosylated p62, C3, PLD3, and NRXN3. Non-AD and AD samples were run on the same gel.

To increase confidence in our automated, large-scale identification of differentially expressed SNO proteins with the *SNO*TRAP probe coupled with MS, we also manually searched the MS database for evidence that the most abundant, unique SNO peptides were present at the correct chromatographic retention time mass-to-charge ratio (*m/z*) [<10 parts per million (ppm)] and that the appropriate product ions were observed in the tandem mass spectra (Fig. 4, A to D, and table S15). For example, SNO sites on p62, PLD3, C3, and NRXN3, despite the presence of many cysteine residues, were found by *N*-ethylmaleimide (NEM) modification to be present predominantly at Cys^44^, Cys^487^, Cys^720^, and Cys^1018^, respectively (Fig. 4, A to D, and table S15).

To confirm the robustness of our proteomics quantification by *SNO*TRAP with MS analysis, we examined the levels of SNO-p62, SNO-C3, SNO-NRXN3, and SNO-PLD3 by immunoblot analysis following enrichment via *SNO*TRAP reagent.Indeed, in accord with our label-free *SNO*TRAP quantification by MS, these immunoblots demonstrated that the levels of S-nitrosylated p62, C3, NRXN3, and PLD3 were substantially increased in AD over the non-AD group (Fig. 4, E to H).

### Role for female-predominant SNO-C3 in synaptic phagocytosis by human microglia

**Fig. 5.**
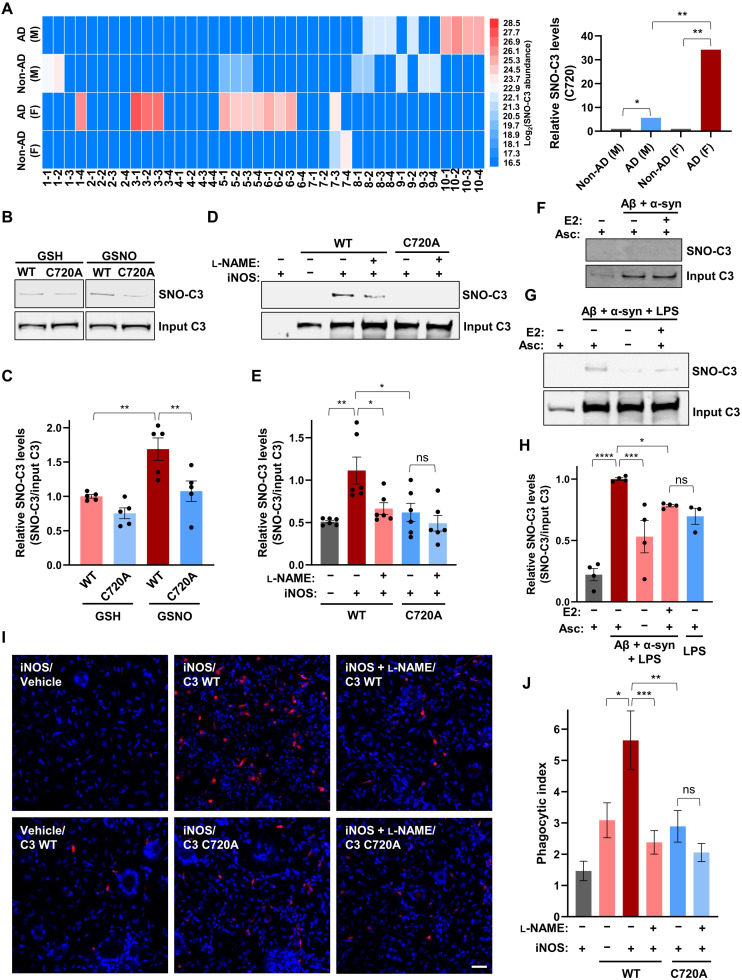
SNO of C3 in female AD brain increases synaptic phagocytosis by human microglia (hiMG). (**A**) Heatmap of SNO-C3(C720) protein (for each replicate of 40 human brains). SNO protein represented by colored box reflecting log_2_ (protein abundance; left panel). SNO protein levels were calculated from MS peaks on using Spectrum Mill software. Histogram shows relative SNO levels of C3 in male AD and female AD compared to respective non-AD brains, as well as female AD compared to male AD (right panel). (**B**) SNO-C3 in WT C3- and mutant C3(C720A)–transfected HEK293T cells after exposure to 50 μM S-nitrosoglutathione (GSNO) or control glutathione (GSH). (**C**) Ratio of SNO-C3/input C3 protein (*n* = 5). (**D**) SNO-C3 in HEK293T cells cotransfected with iNOS plus WT C3 or mutant C3(C720A) in the presence and absence of 1 mM l-NAME. (**E**) Ratio of SNO-C3/input C3 protein (*n* = 6). (**F** and **G**) SNO-C3 in hiMG after exposure to 750 nM Aβ oligomers and 150 nM α-synuclein aggregates, plus LPS (10 ng/ml). Cells were incubated for 18 hours with or without 5 nM 17β-estradiol (E2) pretreatment for 4 hours. (**H**) Ratio of SNO-C3/input C3 protein (*n* = 3 or 4). (**I**) pHrodo-labeled (red) synaptosomes engulfed by hiMG cells after exposure to CM from HEK293 cells cotransfected with iNOS and WT C3 or mutant C3(C720A) in the presence or absence of 1 mM l-NAME. Scale bar, 50 μm. (**J**) Quantification of pHrodo-labeled synaptosome uptake (*n* = 3 randomly selected fields in seven independent experiments with 21 values measured per condition). All histograms represent means ± SEM; *P < 0.05, **P < 0.01, ***P < 0.001, and ****P < 0.0001; ns, not significant, by two-tailed Student’s *t* test (A, right), ANOVA with Tukey’s test (C, E, and J) or Fisher’s least significant difference (LSD) test (**H**) for multiple comparisons.

Complement factor C3, which plays a pivotal role in the innate immune system (*51*), was among the most abundant S-nitrosylated proteins in AD brains. C3 has previously been linked to AD pathology (*52*, *53*) but was not recognized to be S-nitrosylated or to be distributed in a sex-specific manner. In our SNO protein datasets, we observed a significant increase in SNO-C3 in female AD brains, exhibiting a 34.2-fold increase over female non-AD control brains (*P* < 0.03) by Spectrum Mill quantitative analysis, while male AD brains exhibited only a 5.6-fold increase over their respective non-AD brains (*P* < 0.5) (Fig. 5A and table S7). A detailed analysis of our MS datasets revealed for C3 that three cysteine residues could be identified as SNO sites, with Cys^720^ being the predominant site of SNO in AD brains. In addition, Cys^1158^ and Cys^707^ were identified in 1 of 10 female AD samples and 1 of 10 male non-AD samples, respectively (fig. S4). In contrast, the predominant site of SNO occurred on C3 at Cys^720^, particularly in the female AD brain, as detected with greatly increased levels (by Spectrum Mill quantification) of SNO-Cys^720^ in four technical replicates from 5 of 10 female AD samples versus low detection in only a single female non-AD sample (Fig. 5A). Considering male brains, mildly increased levels of SNO-Cys^720^ were detected in 3 of 10 male AD samples and slight increases in 3 of 10 male non-AD samples, implying that age alone could be a factor for SNO-C3 formation in some male brains (Fig. 5A). These findings highlight the sex-based discordance regarding SNO of C3 (SNO-C720) in female versus male AD brains.

C720 is located in the C3a domain, known to be involved in triggering microglial phagocytosis (*54*). We therefore constructed a non-nitrosylatable C3 mutant, in which alanine was substituted for cysteine at position 720, and found significantly decreased SNO by the physiological NO donor, S-nitrosoglutathione (GSNO) (Fig. 5, B and C), and by endogenously generated NO from inducible NOS (iNOS) (Fig. 5, D and E). To study the effects of this nitrosylation event mechanistically, we used a model system of human induced pluripotent stem cell (hiPSC)–derived brain microglia (hiMG) (*55*). In microglia cells, β-estradiol (E2) is known to block generation of NO by inhibiting iNOS expression, therefore preventing, at least in part, lipopolysaccharide (LPS)–induced inflammatory responses (*56*, *57*). We found that physiologically relevant, nanomolar levels of β-estradiol could prevent SNO of C3 induced by Aβ and α-synuclein. For this experiment, we exposed hiMG to Aβ oligomers because they are thought to contribute to the pathology of AD brains, in conjunction with α-synuclein aggregates, which are also found in most AD brains although primarily known for their presence in Parkinson’s disease and Lewy body dementia. In addition to Aβ/α-synuclein, to increase the level of NO further, hiMG were exposed to a low concentration of LPS (LPS or endotoxin) that by itself was not sufficient to produce S-nitrosylated C3, as assessed by biotin-switch assay. LPS was used in this context because infections producing this toxin have been associated with the onset or progression of AD (*56*, *57*). This combination of stimuli induced sufficient SNO-C3 to be detected by the biotin-switch assay, and this formation of SNO-C3 was inhibited by the addition of β-estradiol. As a control, α-estradiol, a nonfeminizing stereoisomer of β-estradiol, did not inhibit SNO-C3 production (Fig. 5, F to H, and fig. S5). These findings imply that β-estradiol may protect females in the premenopausal years from aberrant SNO of C3. As estrogen levels fall after menopause, and particularly in the presence of low-grade infection (e.g., urinary tract infections with *Escherichia coli* producing LPS, which are very common in females), C3 would become S-nitrosylated.

Next, we studied the possible involvement of SNO-C3 in triggering synaptic pruning by microglia since synaptic loss is known to be an important neuropathological correlate of cognitive decline in AD (*58*, *59*). We found that SNO-C3 increased microglial phagocytosis of synaptic membranes (Fig. 5, I and J, and fig. S6). For these experiments, we used conditioned medium (CM) containing SNO-C3 released from iNOS-transfected human embryonic kidney (HEK) 293 cells that were cotransfected with either WT C3 or non-nitrosylatable mutant C3(C720A), as this allows us to selectively compare the effect of SNO-C3 to non-nitrosylatable C3. We observed that CM from cells transfected with WT C3, but not non-nitrosylatable mutant C3, increased phagocytosis of synaptosomal membranes by hiMG, implying causality of the reaction of this cysteine thiol. Moreover, we found that the NOS inhibitor, l-N^G^-nitro arginine methyl ester (l-NAME), blocked both SNO-C3 formation in these HEK293 cells and the effect of C3-containing CM on hiMG phagocytosis of synaptosomes (Fig. 5, I and J). Together, these data are consistent with the notion that generation of endogenous NO (fig. S7) triggers sufficient SNO of C3 to activate in a causal manner phagocytosis and consequent abnormal pruning of synapses, which is known to be mediated by C3 in multiple AD model systems (*54*). Together, these findings show that SNO-C3 triggers phagocytosis of synaptic membranes by hiMG, and this pathway would be expected to be most prominent in postmenopausal human female brains manifesting AD pathology. The production of SNO-C3 in this manner may thus contribute to our understanding of the increased incidence of AD in females over males.

## DISCUSSION

Prior work has linked SNO proteins in neurons and glial cells to neurodegenerative diseases, including AD (*22*, *24*, *60*, *61*). These S-nitrosylated proteins often exist at low abundance, and coverage was fairly sparse in these previous studies, with at most on the order of a couple of hundred SNO proteins detected. A key concept is that high coverage of the proteome is extremely important to find these proteins and the pathways/reactomes that they are involved in. Our deep, rigorous SNO-proteome analyses using the sensitive *SNO*TRAP probe revealed highly reproducible lists of altered key SNO proteins and SNO sites in both male and female AD human brains compared to their respective non-AD controls and allowed us to prioritize molecular alterations on the basis of multiple datasets with label-free quantification. Bioinformatic analysis of the data provided previously unrecognized insights into proteins and pathways that may be associated with AD. The acquisition of rich proteome informatics depends on (i) access to high-quality, postmortem human AD brain tissue and non-AD brain tissue (*62*), together with (ii) an MS-based proteomics platform, including nanoLC coupled with high-resolution MS/MS analysis (*63*), and (iii) the modified SNO-protein enrichment method described here. Various NOS isoforms have been shown to be elevated in human AD brains (*42*), in part accounting for the excessive levels of reactive nitrogen species/NO in comparison with non-AD brains (including normal brains) and contributing to aberrant SNO of several key proteins. Oxidative/nitrosative stress and inflammatory reactions are thought to be major driving forces in the pathogenesis of degenerative disease, thus implicating aberrant protein SNO in the etiological process (*24*, *64*).

In this study, our modified *SNO*TRAP method, which identified SNO sites on proteins, ensured high specificity (98%) and technical reproducibility (RSD < 20%). The intrinsic value of identifying the cysteines that have been substituted in this fashion is that it allows speculation, hypothesis, and future experimentation on the effect of this PTM on individual protein function and evaluation of network effects. Using this approach, we analyzed 40 human brain samples, which led to the discovery of nearly 1500 SNO proteins and almost 3000 SNO sites. At present, development of therapeutic treatments for AD is in part hampered by a lack of sensitive and specific biomarkers and targets for disease progression (*65*). Therapeutic target and biomarker discovery and validation remain a critical research interest (*66*–*68*). p62, PLD, and C3 have all been implicated in neurodegenerative diseases in general and in AD in particular (*52*, *69*–*72*), each of which we found to be S-nitrosylated in AD brains over controls. Our data represent the first indication that protein SNO may potentially affect the function of these proteins in the context of the human AD brain.

A major SNO-modified protein found in AD brain is p62, a multifunctional protein involved in protein turnover (via both macroautophagy and the proteasome), oxidative stress, and other cell functions (*73*, *74*). In addition, p62 has been found in protein inclusions of several neurodegenerative disorders (*69*, *75*). Oxidative damage to the promoter region of p62 has been reported in neurodegenerative diseases and may account for decreased expression of p62, which may contribute to neurodegeneration (*76*, *77*). Decreased levels of p62 protein might disturb signaling pathways, including nuclear factor кB and Nrf2, thus increase oxidative stress, and impair neuronal survival (*73*). In our dataset, we found p62 to be S-nitrosylated at Cys^44^ in the Phox and Bem1 (PB1) domain. Through this domain, p62 interacts with several kinases, including the atypical protein kinase C (PKC) subfamily of enzymes, PKCζ and PKCι (*69*, *73*). These atypical PKCs have been linked to aberrant Aβ peptide and phospho-tau expression in AD brain (*78*). Therefore, PTM sites in the PB1 domain of p62, such as that for SNO, could represent a previously undiscovered therapeutic target for AD. In addition, the microtubule-associated protein 1A/B light chain 3 (LC3)-interacting region of p62 is involved in macroautophagy. Along these lines, we recently found that SNO of an additional cysteine in this domain can inhibit autophagic flux, thus preventing the clearance of misfolded proteins to contribute to the pathogenesis of neurodegenerative disorders (*79*).

Another enriched SNO protein in AD brains, PLD3, is a type 2 endoplasmic reticulum–associated transmembrane protein that is highly expressed in the brain, mainly in neurons, and may function in the endosomal-lysosomal system (*80*–*82*). Rare coding variants in PLD3 may confer increased risk for AD (*70*, *83*), although the exact role of PLD in the disease remains unknown (*84*, *85*). In our dataset, we observed SNO of PLD3 at Cys^487^ in samples from both male and female AD brains. These results may suggest future avenues for investigating the association of PLD3 to AD.

Notably, SNO proteins associated with complement pathways are enriched in both male and female AD brains. However, SNO of C3, representing the point of convergence of the various complement cascades, was detected predominantly in female AD brains. This is of potential importance in the pathogenesis of AD as complement pathways have also been linked to neuroinflammation and tau pathology (*86*). Moreover, as part of the innate immune system, the complement cascade is known to be involved in clearance of both pathogens and damaged cells but also functions with microglia for normal elimination of excessive synapses during neurodevelopment (*87*). Critically, this process has recently been linked to AD through mouse models with direct implications for synapse loss in the human AD brain (*54*, *88*). For example, complement proteins were shown to be activated in AD to mediate pruning of synapses by microglia, thus contributing to synaptic loss. We found evidence for SNO of the complement-related proteins C3, F2, and CFH, which may affect their biological activity. Critically, one site on C3 (Cys^720^) was differentially nitrosylated in female over male AD brains. We found evidence that SNO of this site in the C3a domain triggered increased microglial phagocytosis of synaptic membranes, emulating a critical pathological feature of AD (*58*, *89*). Emerging evidence suggests that various risk factors contribute to the predominance of AD in females over males. These factors include genetic risk, for example, with the apolipoprotein E4 (APOE4) allele found to be increased in females over males; comorbid risk of medical conditions such as cardiovascular disease, diabetes, and chronic inflammation; and hormonal risk affecting thyroid disease, pregnancy, and menopause, etc. (*90*). Accordingly, we hypothesized that menopause-associated up-regulation of inflammation in the brain could be causally linked to the aberrant increase in SNO-C3 that we observed. Together with prior findings, we found evidence that a drop in β-estradiol levels leads to increased iNOS expression, thus contribution to SNO-C3 production. Thus, dysregulation of the complement system may play a role in the pathogenesis of AD and explain, at least in part, the female predominance of the disease. While the in vitro trigger of phagocytotic activity in hiMG by SNO-C3 is consistent with the notion that SNO-C3 stimulates abnormal phagocytosis of synapses in vivo (*50*), we note that our experiments conducted in vitro with hiMG need to be interpreted cautiously considering the fact that we demonstrate the presence of SNO-C3 in human AD brain, but not specifically on human microglia in those brains.

In addition, during our analysis of differentially expressed SNO proteins by sex in human AD brains with GO and RCTM pathway analysis for biological processes, we found other potentially important pathogenic pathways affected in AD brain. For example, in male AD brains, we observed significant enrichment in SNO proteins associated with transsynaptic signaling and synaptic transmission (*P* = 5.0 × 10^−8^). This is interesting given the importance of synaptic loss to cognitive decline in AD (*58*, *89*). Extensive studies have also supported a direct relationship between oxidative stress and synaptic dysfunction in AD (*59*). In female AD brains, we found that pathways involved in the respiratory electron transport chain (*P* = 1.7 × 10^−7^), which is strongly correlated to oxidative stress, were significantly enriched with SNO proteins (*91*).

In summary, the present study systematically profiles protein SNO in postmortem human brains from male AD and female AD versus male non-AD and female non-AD control samples. Our semiquantitative proteomic analysis provides a substantive human brain S-nitrosoproteome database for future work. We have identified various enriched SNO proteins in male and female AD samples compared to their respective non-AD controls, which may serve as clues for pathogenic pathways in AD and therefore as previously undiscovered therapeutic targets. Our GO and pathway analyses suggest that altered protein SNO is involved in several key biological processes that have been previously linked to AD and suggest pathways for further investigation. Last, our data suggest a unique mechanism by which protein SNO modulates complement (C3) activity in a sex-dependent manner, thereby providing a molecular link between NO signaling and the complement cascade in AD pathogenesis.

## MATERIALS AND METHODS

### Reagents

Protease cocktail inhibitor, iodoacetamide (IAM), and Hepes were purchased from Sigma-Aldrich. The following reagents were bought from Pierce: high-capacity streptavidin agarose resin, bovine serum albumin (BSA) digest standard, and peptide desalting spin columns. NEM was from Thermo Fisher Scientific. Vivaspin Turbo 4 filters with a nominal molecular weight cutoff (MWCO) of 10 kD were bought from Thermo Fisher Scientific. Biotin–polyethylene glycol 3–propionic acid was purchased from ChemPep Inc. Sequencing grademodified trypsin was provided by Promega. Acetonitrile (ACN) with 0.1% (v/v) formic acid (FA) and water with 0.1% (v/v) FA for nanoLC-MS analysis were purchased from MilliporeSigma and Honeywell Research Chemicals, respectively.

Primary antibodies were purchased as follows: anti–glyceraldehyde-3-phosphate dehydrogenase antibody (Cell Signaling Technology, 2118S; 1/1000 dilution), anti-PLD3 antibody (Abcam, ab76433; 1/500 dilution), anti-p62 (SQSTM1) antibody (Abcam, ab207305; 1/1000 dilution), anti-NRXN3 antibody (MilliporeSigma, ABN96; 1/1000 dilution), and anti-C3 antibody (Cell Signaling Technology, 97425; 1/1000 dilution; Abcam, ab200999; 1/3000 dilution). Secondary antibodies were bought as follows: goat anti-rabbit immunoglobulin G H&L (Abcam, ab6721, 1/7000 dilution) or IR-dye 800CW-conjugated goat anti-rabbit (1,15,000, Li-Cor, 926-32211). The human C3 gene open reading frame complementary DNA clone expression plasmid (pCMV-C3) was purchased from Sino Biological, and C720A mutant C3 constructs were generated using the QuikChange Lightning Multi Site-Directed Mutagenesis Kit (Agilent Technologies, 210514), according to the manufacturer’s protocol.

### Human stem cell–derived microglia and cell lines

hiPSC cultures and microglial differentiation methods were as previously described (*54*). Fully differentiated microglial cells were placed into phenol-free Iscove’s modified Dulbecco’s medium (IMDM; Thermo Fisher Scientific, 21056023); 1 day later, cells were pretreated with 5 nM β-estradiol (Sigma-Aldrich, E8875) or control diluent for 4 hours and then exposed to 750 nM oligomerized Aβ (Anaspec, AS-20276), 150 nM oligomerized α-synuclein (Anaspec, AS-20276), and LPS (10 ng/ml; Invivogen, tlrl-3pelps) were incubated in the presence or absence of β-estradiol as previously described (*55*, *92*, *93*). For transient transfection of HEK293T cells (System Biosciences, LV900A-1) with plasmids encoding either WT C3 or mutant C3(C720A), Lipofectamine 2000 was used according to the manufacturer’s instructions (Thermo Fisher Scientific, 11668019).

### Synthesis of *SNO*TRAP-biotin

A mixture of 50 mg of biotin-peg3-proprionic acid (1 eq., 0.11 mmol), 34 μl of diisopropylcarbodiimide (2 eq., 0.22 mmol), and 39 mg of *N*-hydroxysuccinimide (NHS; 3 eq., 0.33 mmol) was dissolved into 5 ml of dichloromethane (DCM) under argon. The reaction was stirred at room temperature (RT) overnight. To purify the product NHS-ester, the mixture was separated on Biotage SNAP Cartridges (C18, 10 g) with a linear gradient starting from DCM:MeOH (99:1, v/v) for 5 min to DCM:MeOH (80:20, v/v) over 15 min at a flow rate of 12 ml/min by flash chromatography (Biotage Isolera Prime, USA). To a stirred solution of 58 mg of NHS-ester (1 eq., 0.107 mmol) in DCM (5 ml), we added 35 mg of phosphothiol (1.1 eq., 0.177 mmol) and 17 ul of triethylamine (1 eq., 0.107 mmol) successively under argon. The reaction was stirred at RT overnight. The resulting clear solution was then concentrated under reduced pressure and purified by flash chromatography with a linear gradient starting at 100% DCM for 5 min to DCM:MeOH (75:25, v/v) over 20 min to give the desired product (yield around 40%). Nuclear magnetic resonance and high-resolution MS analyses were described previously in detail (*39*). The product was dried down, aliquoted into 25 mM stock solutions in dry ACN, and frozen for future use.

### Preparation of aggregated α-synuclein and oligomeric Aβ

α-Synuclein was prepared as previously described (*92*). In brief, endotoxin-free recombinant human α-synuclein protein (Anaspec, #AS-55555-1000) was dissolved in high-performance LC–grade water (Sigma-Aldrich, #270733) at a concentration of 200 μM. For aggregation of α-synuclein, samples were placed in a thermomixer, shaken continuously at 1400 rpm for 6 days at 37°C, and centrifuged at 1000*g*, and the pellet was aliquoted and frozen at −80°C. For preparation of Aβ oligomers, Aβ_1–42_ peptide (Anaspec, #AS-20276) was prepared as previously described (*93*). Briefly, Aβ was suspended in hexafluoroisopropanol to a concentration of 1 mM, and solvent was evaporated at RT overnight. Peptide was resuspended in dry dimethyl sulfoxide to a final stock concentration of 5 mM, sonicated at RT for 10 min, diluted 10-fold in minimum essential medium (GIBCO), and then incubated at 4°C for ≥24 hours. The percentage of soluble oligomers used in the experiments was 15% by dynamic light scattering and other chemical assessments, as previously described (*93*).

### Human brain tissue samples

The study included autopsy-confirmed human brains with AD (*n* = 10 females and *n* = 10 males) and non-AD (*n* = 10 females and *n* = 10 males), matched for age, sex, education, and ethnicity. The tissues from the prefrontal cortex (Brodmann area 10, a region of the brain affected in these cases of AD, were analyzed. All brain tissues were obtained from the University of California Medical Center and the San Diego VA Medical Center Brain Bank and were flash-frozen at postmortem examination. The study was approved by the local Ethics Committee of both medical centers. Dementia diagnoses were performed independently by two experienced clinicians in alignment with the consensus criteria for AD (*3*). All AD brains fulfilled Braak stage 5 or 6, while the non-AD fulfilled Braak stage 0, 1, or 2. Table S1 represents the clinical and demographic characteristics of the samples. All the tissues were stored at −80°C until use.

### Preparation of brain tissues for MS

Tissues were homogenized into lysis buffer [100 mM Hepes-NaOH (pH 7.7), 1 mM EDTA, 0.1 mM neocuproine, 1% Triton X-100, 20 mM IAM, 1% protease inhibitor cocktail, and 0.1% SDS] freshly prepared on ice using a Teflon pestle and a Jumbo Stirrer (Thermo Fisher Scientific). The homogenates were centrifuged at 16,000*g* for 15 min at 4°C, and supernatants were collected. The protein concentration was determined by the Bradford assay. The supernatants were washed once with 1 volume of 50 mM Hepes (pH 7.7) by centrifugation at 5000*g* for 25 min at 4°C with 10-K MWCO spin filters.

*SNO*TRAP-labeling stock solutions (in ACN) were added to all samples to reach a final concentration of 1.5 mM to selectively convert SNO to stable disulfide-iminophosphorane. Negative controls were generated by doping of the same volume of 40% ACN in 50 mM Hepes (pH 7.7). The mixture was incubated at RT for 2 hours. After reaction, excessive reagents were removed by three washes with 50 mM Hepes (pH 7.7) buffer with 10-K filters, followed by trypsin digestion at 37°C overnight.

After digestion, 200 μl of prerinsed high-capacity streptavidin agarose beads were added to each sample and incubated for 2 hours at RT with gentle shaking. To decrease nonspecific binding, the beads were washed twice with fivefold volumes of the following five buffers successively: buffer I [100 mM ammonium bicarbonate (ABC), 150 mM NaCl, and 1 mM EDTA (pH 7.4), containing 0.05% SDS and 0.1% Triton X-100)], buffer II [100 mM ABC, 150 mM NaCl, and 1 mM EDTA (pH 7.4) containing 0.1% SDS], buffer III (100 mM ABC, 0.05% SDS, and 150 mM NaCl), buffer IV (100 mM ABC), and buffer V (50 mM Hepes, pH 7.7).

The bound peptides were eluted with 10 mM tris(2-carboxyethyl)phosphine [TCEP; in 50 mM Hepes (pH 7.7)] and then alkylated with 100 mM NEM for 2 hours at RT. After alkylation, samples were desalted with Pierce C18 spin columns and stored at −80°C pending analysis. Sample preparation and storage were conducted in the dark.

### NanoLC-MS analysis

The desalted peptides, dissolved in 0.1% FA, were analyzed using a Q Exactive mass spectrometer coupled to Easy-nLC 1000 (Thermo Fisher Scientific, Waltham, MA) interfaced via a nanoSpray Flex ion source. At least four technical runs were conducted for each biological replicate, i.e., from tissue of a single individual. FAs in water (0.1%) and in ACN (0.1%) were used as mobile phases A and B, respectively. Aliquots (3 μl) of the samples were injected onto a C18 precolumn (75 μm ID × 20 mm, 3 μm; Thermo Fisher Scientific) and separated by a C18 column (75 μm ID × 250 mm, 2 μm; Thermo Fisher Scientific) with a stepwise gradient (1% B for 10 min, 1 to 60% B for 110 min, and then 60 to 100% B for 10 min) followed by a 10-min post-run at 1% B at a flow rate of 300 nl/min. Mass spectra were acquired in data-dependent mode using the following settings: spray voltage, 2.2 kV; capillary temperature, 250°C; S-lens RF level, 60%; no sheath and auxiliary gas flow; resolution at 70,000; scan range of 350 to 1800 Th. The 10 most abundant ions with multiple charge states were selected for fragmentation with an isolation window of 2 Th and a normalized collision energy of 28% at a resolution of 35,000. The maximum ion injection times for the full MS scan and the MS/MS scan were both 100 ms. The ion target values for the full scan and the MS/MS scans were set to 3 × 10^6^ and 1 × 10^5^, respectively. Xcalibur software was used for data acquisition.

### MS data processing

Agilent Spectrum Mill MS proteomics Workbench B.06 was used for peak list generation, database searching, label-free semiquantitative assessment, and FDR estimation. Parameters for data extractions were as follows: precursor MH^+^ of 300 to 8000 Da, scan time range of 0 to 200 min, sequence tag length > 1, merge scans with same precursor *m/z* ±30 s and ±0.05 *m/z*, default for precursor charge, true for find ^12^C precursor, and MS noise threshold 100 counts. MS/MS spectra were searched against the human SwissProt protein database (downloaded on 18 June 2019) with ±10-ppm precursor ion tolerance and ±20-ppm fragment ion tolerance. The search included variable modifications of methionine oxidation, protein N-terminal acetylation, deamidation of asparagine, and fixed modification of NEM on Cys. For both peptide identification and protein polishing, the FDR was set to 1%. Peptide identifications were accepted only if the following confidence thresholds were met: peptide score > 5 and scored peak intensity > 70%. Minimal peptide length was set to five amino acids, and a maximum of two missed cleavages was allowed. The MS/MS spectra were inspected manually to validate the peptide/protein identifications. In addition, for proteins detected in one group, but not another, the raw LC-MS data files for the latter were searched manually for the presence of protein-related peptides detected in the former. The MS raw data have been deposited in the ProteomeXchange Consortium (http://proteomecentral.proteomexchange.org) via the PRoteomics IDEntifications (PRIDE) partner repository with the dataset identifier < PXD020945 >.

### General analytical approach to assess protein SNO

All samples were treated identically and analyzed in a masked fashion. Our previous work on the mouse (*23*, *39*) had suggested that, in disease, the SNO-proteome was reprogrammed, rather than adding newly generated SNO proteins onto the endogenous normal proteome. This would in turn suggest that selective protein denitrosylation is relevant in addition to de novo SMO, and it would therefore be important to look at the entire SNO-proteome across disease and normal conditions, as performed here.

Each tissue sample was weighed, the protein content was measured, and then a similar amount of protein was processed for each sample. To maximize the discovery of SNO proteins, we carried out four technical replicates to analyze as large a fraction of the proteome as possible to make fair comparisons across samples (*23*). The results of the technical replicates were combined to provide a list of discovered proteins. Using this approach, all tissue samples yielded similar numbers of proteins. The method detects SNO peptides, and some proteins have more than one SNO site. Therefore, the data were processed through Spectrum Mill for protein identification (ID), and semiquantitative comparisons of samples were made on the basis of protein IDs rather than SNO sites. Otherwise, bias would be introduced that favored proteins with more than one SNO site. The curated SNO proteome for each sample was then combined with all of the samples in a class, yielding 10 samples for AD brain of each sex to compare to that of age- and sex-matched non-AD control brains (total of 40 samples), but we also retained each sample’s SNO proteome separately to determine how often a specific protein occurred in tissue samples for each condition to insure the reliability and reproducibility of the data.

To determine overall changes, we used Venn diagrams to present all of the data in a compartmentalized fashion to reveal global changes in the S-nitrosoproteome and the specific changes that underlie these global changes. A Venn diagram that compares two sets of data has three compartments; hence, for our results, that would be one compartment for AD, one for non-AD, and a third that has the proteins that appear in both AD and non-AD brains. Note that a protein that appears only in the AD compartment means that it was undetectable in the non-AD using the criteria described here. It would therefore be uniquely S-nitrosylated in AD within our limits of detection, and the ratio of the protein in AD to non-AD would be infinite. The SNO proteins that appear in the third compartment, present in both AD and non-AD, were analyzed in Volcano plots and demonstrated that some proteins were modified more significantly in AD versus non-AD or vice versa. Some of these proteins were selected for semiquantitative analysis of their mass spectra by spectral counting using Spectrum Mill software.

### Western blots and biotin-switch assay for SNO protein

For immunoblotting, each biological replicate of human frontal cortex represented a pooled homogenate from all samples of that group. Brain tissue was homogenized in ice-cold radioimmunoprecipitation assay (RIPA) buffer (2 ml/g) and centrifuged at 14,000*g* for 15 min at 4°C. Protein concentration was determined with the Bradford assay, using BSA as a standard. The lysate was subjected to tris-HCl 4 to 20% precast linear gradient gels (Bio-Rad, 4561093). Samples were transferred from the gel to polyvinylidene difluoride membrane (Bio-Rad, 1620174). The membranes were blocked with 5% milk in phosphate-buffered saline (PBS) 0.05% Tween 20 for 1 hour at RT and incubated with primary antibody overnight at 4°C. After five washes with tris-buffered saline 0.05% Tween 20, the membrane was incubated with a horseradish peroxidase–conjugated secondary antibody for 1 hour at RT. Protein bands were processed with electrochemiluminescence reagent for 2 min and visualized using FluorChem 8900 from Alpha Innotech. ImageJ software was used to quantify each signal, and quantified results were normalized to loading controls. Experiments were performed in a blinded fashion. All data represent at least three independent experiments, presented as mean and SEM.

To detect SNO proteins by immunoblot in addition to mass spectrometric analysis, the pooled 20 AD human brain lysates or 20 non-AD lysates were labeled with *SNO*TRAP, pulled down by streptavidin agarose resin, and eluted with TCEP as described above. The eluted SNO proteins were dried by continuous vacuum sublimation and dissolved in RIPA buffer, and immunoblot was performed as described above.

To monitor SNO-C3 in samples from hiPSC-derived microglial cells or from HEK293T cells, cell lysates were incubated in 10 mM methyl methanethiosulfonate (Sigma-Aldrich, 208795) to block free cysteine residues, exposed to ascorbate (20 mM) to reduce SNO, and then labeled with biotin-HPDP (Dojindo, SB17-10). Biotinylated proteins were pulled down with high-capacity NeutrAvidin-agarose beads (Thermo Fisher Scientific, 29202) and analyzed by immunoblotting with anti-C3 antibodies to identify S-nitrosylated C3.

### Detection of NO-related species by Griess assay

CM from cultures was subjected to the Griess assay using the Nitrate/Nitrite Colorimetric Assay Kit (Cayman Chemical, 780001) following the manufacturer’s protocol. The absorbance of the samples was measured at 540-nm wavelength using a SpectraMax M3 plate reader (Molecular Devices).

### pHrodo-synaptosome phagocytosis assay with hiMG

To generate synaptosomes, the frontal cortex of rat brain was homogenized with Syn-PER synaptic protein extraction reagent (Thermo Fisher Scientific, 87793) on ice with 10 slow strokes. Then, the homogenates were centrifuged at 1200*g* for 10 min at 4°C, and the supernatants were subsequently centrifuged at 15,000*g* for 20 min at 4°C. The pellets were resuspended with Syn-PER synaptic protein extraction reagent following the manufacturer’s protocol. To conjugate pHrodo Red, succinimidyl ester (pHrodo Red, SE; Thermo Fisher Scientific, P36600) to synaptosomes, the isolated synaptosomes were mixed with pHrodo red dye at one-half of the weight of the synaptosomes, mixed with an equal volume of 100 mM sodium carbonate buffer (90 mM Na₂CO₃ and 10 mM NaHCO₃ in PBS buffer), and incubated for 1 hour at RT. Next, an equal volume of PBS was added to the mixture, and the resulting pHrodo-synaptosome conjugates were centrifuged at 15,000*g* for 20 min at 4°C. The pellets were resuspended in phenol red–free IMDM medium to bring to a final concentration of 1 μg/ml (*94*).

For microglial phagocytosis assays of synaptosomes, we used hiMG cells, prepared as previously described (*55*). Briefly, after a 21-day differentiation protocol, hiMG derived from hiPSCs were replated at a density of 1.0 × 10^6^ cells per well on coverslips. One or 2 days later, the culture medium was changed to phenol red–free IMDM, and cells were allowed to recover for 1 day. Then, hiMG cells were incubated for 2 hours in CM derived from HEK293 cells that had been transiently transfected with iNOS and WT or C720A mutant C3 plasmids in the presence or absence of 1 mM l-NAME. Subsequently, pHrodo synaptosomes (5 μg per well) were exposed for 1 hour to hiMG, which were then fixed in 4% paraformaldehyde for 15 min at RT and stained with Hoechst 33342 for 15 min to visualize nuclei. Engulfed pHrodo synaptosomes were imaged with a confocal microscope (Nikon Eclipse Ti2). Three randomly selected fields were analyzed in a masked fashion per condition using Fiji ImageJ2 software. The phagocytic index represents the area of uptake of pHrodo-labeled synaptosomes divided by total cell area.

### Bioinformatics

To analyze functional enrichment of GO processes and pathways, we uploaded the SNO protein IDs into MetaCore software (version 19.4 build 69900). Reactome pathway analysis was performed using STRING (version 11.0) (http://string-db.org). High-reliability interactions (score > 0.7) were kept. To visualize protein-protein interactions, we used Cytoscape plug-in software (version 3.7.2). FDR values below 0.1 were accepted for the analyses using the aforementioned software platforms.

### Statistics

For statistical analyses using label-free quantification of the S-nitrosoproteome, the precursor intensities of SNO proteins, which were estimated in Spectrum Mill by combining precursor intensities of the constituent peptides in MS1 spectra, were used to calculate FC quantitation of the common proteins detected in AD and non-AD groups, with FC defined as total ion intensity of AD divided by total ion intensity of respective non-AD samples. Statistical evaluation of nanoLC-MS label-free differential data was performed after the application of data imputation to reduce the number of missing values. Missing values were replaced by this value according to the local minimum method (*95*). An FC of >2 was considered as increased expression, and an FC of <0.5 was considered as decreased expression and corresponded to a *P* value of <0.05 by Student’s *t* test, as we have described previously (*39*).

For presentation, the number of replicates or experiments is indicated in the individual figure legends. Data are expressed as means ± SEM. Differences between experimental groups were evaluated using an analysis of variance (ANOVA) followed by a post hoc Tukey’s or Fisher’s least significant difference test for multiple comparisons or a two-tailed Student’s *t* test for comparison of two groups, with a difference of at least *P* < 0.05 considered statistically significant.

## Notes

View/request a protocol for this paper from *Bio-protocol*.
