# Supplementary material for: Mechanistic insight into female predominance in Alzheimer’s disease based on aberrant protein S-nitrosylation of C3
Source: Sci Adv. 2022 Dec 14;8(50):eade0764. doi: 10.1126/sciadv.ade0764 (PMC9750152; doi:10.1126/sciadv.ade0764)
Supplement: Supplementary file 1 — Figs. S1 to S7 Tables S1, S2 and S15 [file sciadv.ade0764_sm.pdf]

Science Advances
AAAS

# Supplementary Materials for

## Mechanistic insight into female predominance in Alzheimer's disease based on aberrant protein S-nitrosylation of C3

Hongmei Yang *et al.*

Corresponding author: Steven R. Tannenbaum, srt@mit.edu; Stuart A. Lipton, slipton@scripps.edu

*Sci. Adv.* **8**, eade0764 (2022)
DOI: 10.1126/sciadv.ade0764

**The PDF file includes:**

Figs. S1 to S7
Tables S1, S2 and S15
Legends for tables S3 to S14

**Other Supplementary Material for this manuscript includes the following:**

Tables S3 to S14

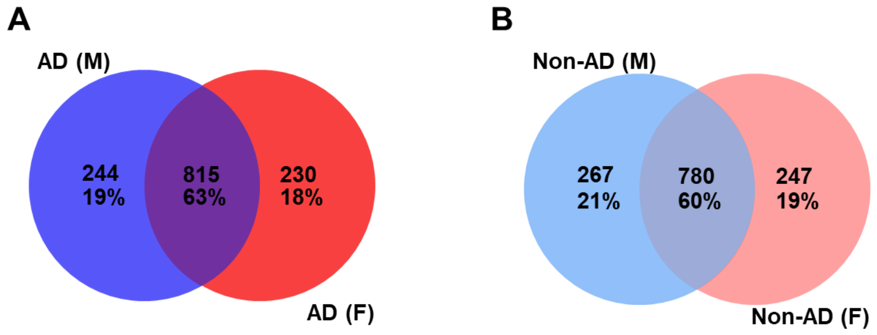


**Fig. S1. Characterization of SNO-proteins in male (M) vs. female (F) brains by SNO-proteomics.** (**A** and **B**) Venn diagrams representing the number of SNO-proteins identified in male AD and female AD brains, and in male non-AD and female non-AD control brains.

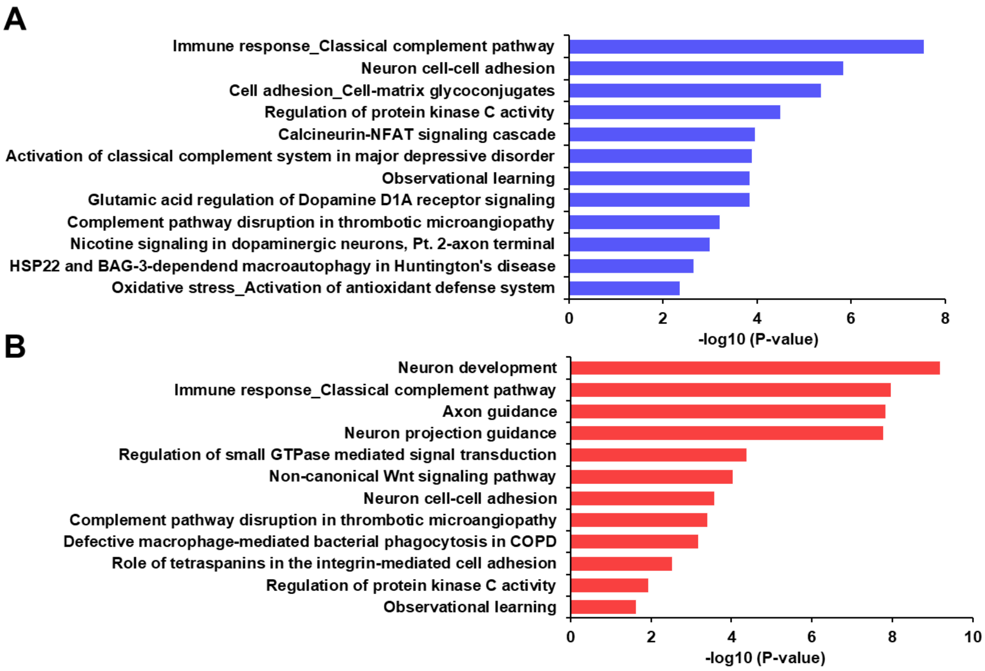


**Fig. S2. GO and pathway analysis of the up-regulated SNO-proteins.** (**A**) GO terms and pathways exclusive to male AD brains compared to control male non-AD brains. (**B**) GO terms and pathways exclusive to female AD brains compared to control female non-AD brains.

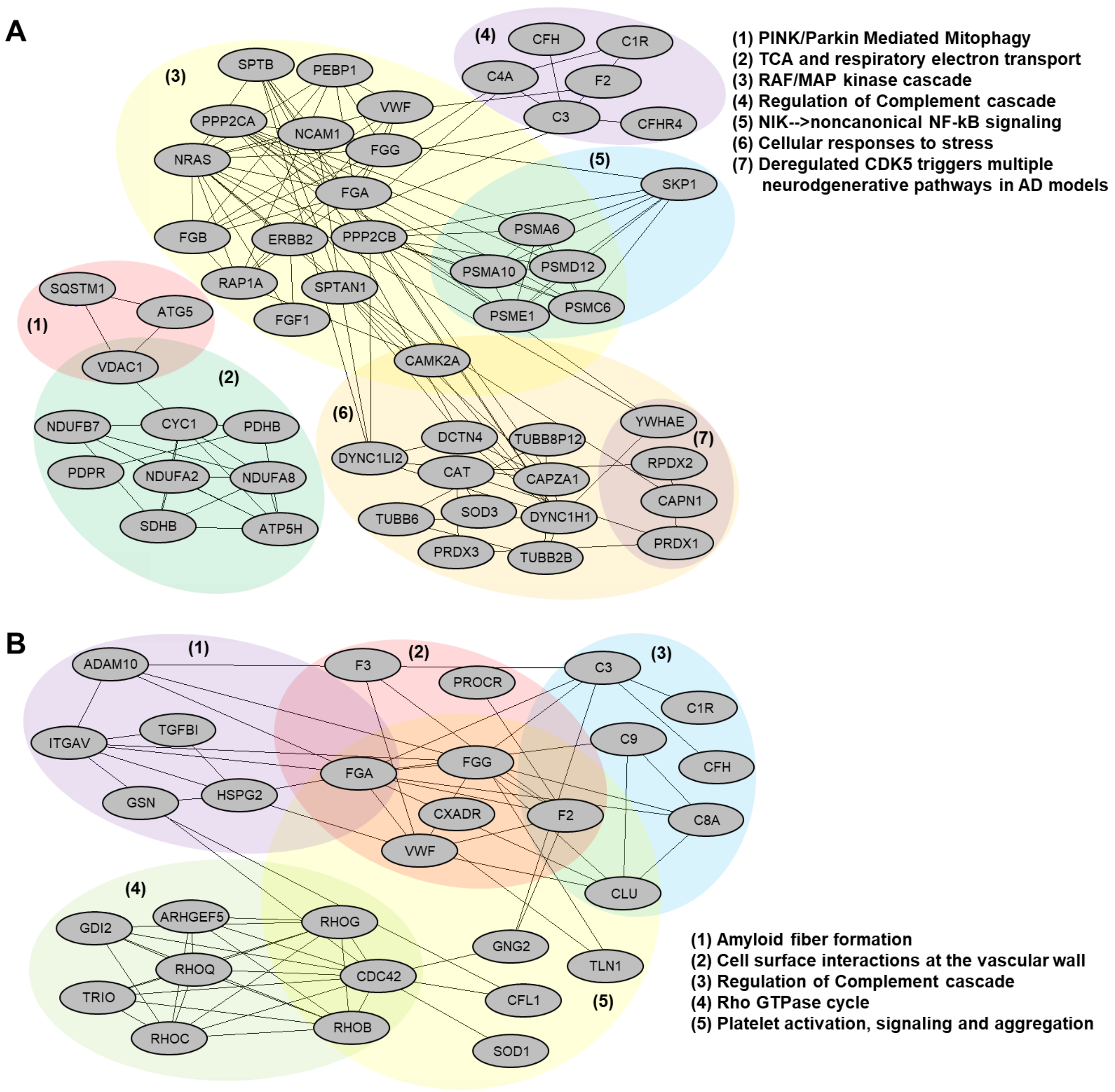


**Fig. S3. Interaction network of up-regulated SNO-proteins by sex.** (**A**) SNO-proteins found exclusively in male AD brains compared to control male non-AD brains. (**B**) SNO-proteins found exclusively in female AD brains compared to control female non-AD brains. Regulated functional protein clusters and complexes are indicated by shadowing.

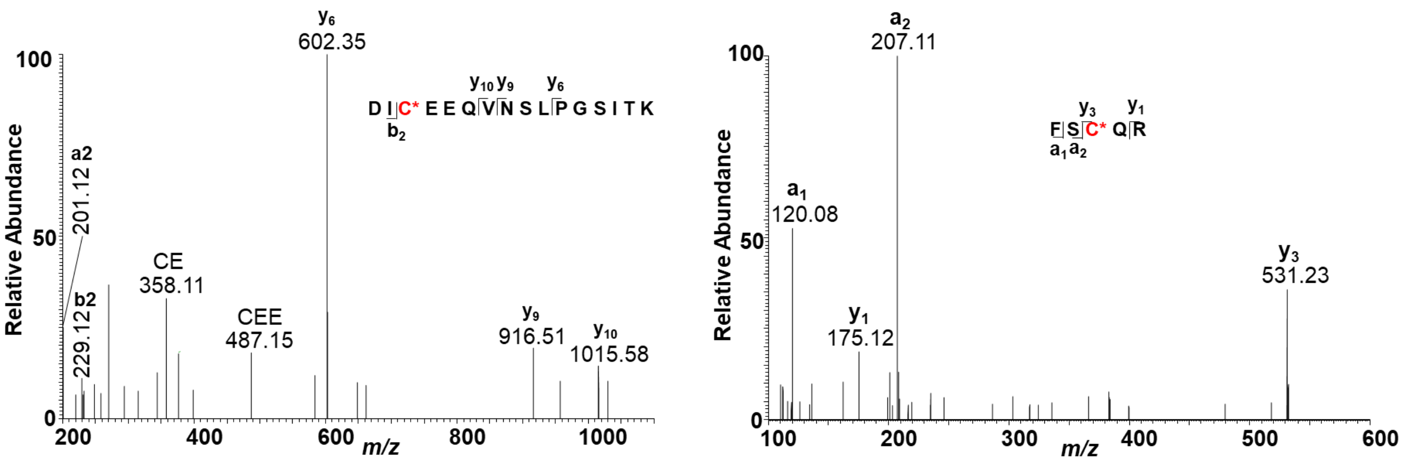


| Fragment ions | Theoretical *m/z* | Observed *m/z* | Δ ppm |
|---|---|---|---|
| DI ($a_2$) | 201.1239 | 201.1230 | -4.5 |
| DI ($b_2$) | 229.1188 | 229.1186 | 0.9 |
| C*E | 358.1072 | 358.1070 | -0.6 |
| C*EE | 487.1498 | 487.1503 | 1.0 |
| PGSITK ($y_6$) | 602.3513 | 602.3500 | -2.2 |
| NSLPGSITK ($y_9$) | 916.5103 | 916.5104 | 0.1 |
| VNSLPGSITK ($y_{10}$) | 1015.5787 | 1015.5801 | 1.4 |

| Fragment ions | Theoretical *m/z* | Observed *m/z* | Δ ppm |
|---|---|---|---|
| F ($a_1$) | 120.0813 | 120.0806 | -5.8 |
| R ($y_1$) | 175.1195 | 175.1187 | -4.6 |
| FS ($a_2$) | 207.1133 | 207.1124 | -4.3 |
| C*QR ($y_3$) | 531.2349 | 531.2325 | -4.5 |

**Fig. S4. Tandem mass spectrum of C3 peptides showing sites of S-nitrosylation**. (**A**) S-Nitrosylation of C3 at Cys1158 in female AD brain. (**B**) S-Nitrosylation of C3 at Cys707 in male non-AD brain. The a-, b-, and y-type product ions are indicated. Summary of SNO-peptide fragments includes calculated monoisotopic masses (mass) and accuracy of mass measurements in parts per million (ppm). Asterisks indicate modification sites.

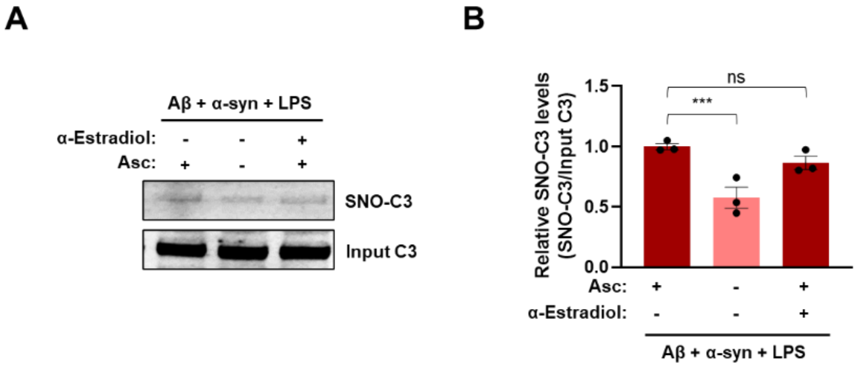


**Fig. S5. Unlike β-estradiol, α-estradiol does not inhibit SNO-C3 production in hiPSC-derived microglial cells (hiMG).** (**A**) Representative immunoblots of SNO-C3 after exposure of hiMG to 750 nM oligomerized Aβ/150 nM α-syn plus 10 ng/ml lipopolysaccharide (LPS). Cells were incubated for 20 hours after a 4-hour pretreatment with 5 nM 17α-estradiol, or control diluent. (**B**) Histogram shows ratio of SNO-C3/input C3 protein. Values are mean ± SEM. Statistical significance evaluated by ANOVA with post hoc Fisher's LSD test. ***$P < 0.001$; ns: not significant, $n = 3$ biological replicates.

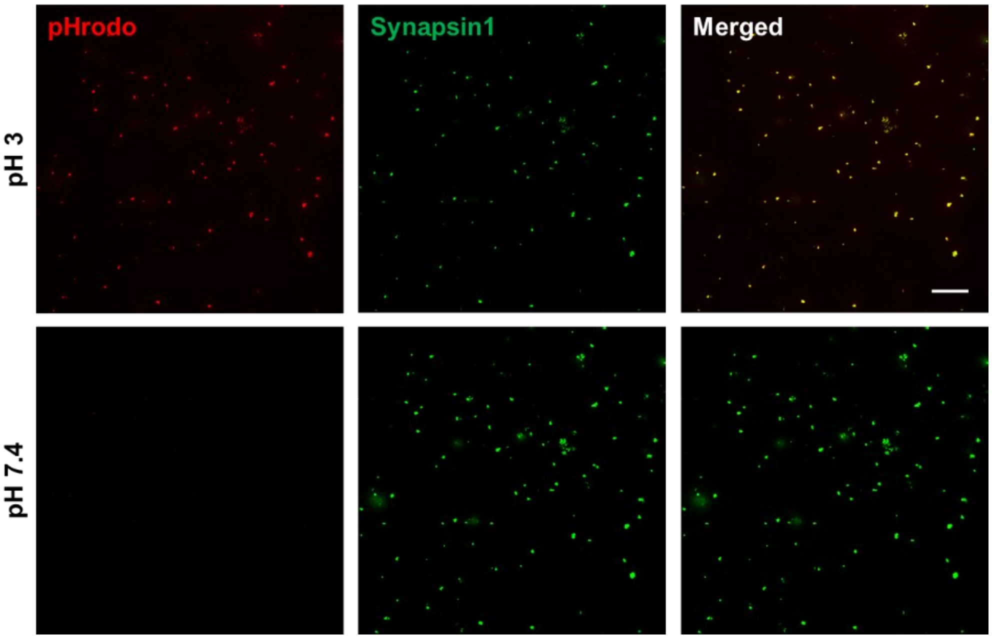


**Fig. S6. pHrodo-labeled synaptosomes.** Representative images of pHrodo (red)-labeled synaptosomes. pHrodo-labeled synaptosomes were immunostained with presynaptic marker anti-Synapsin1. Each image was acquired in pH 3 or pH 7 in PBS buffer. Scale bar: 50 µm.

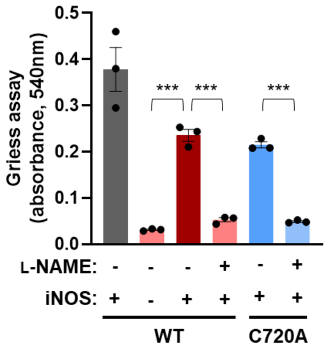


**Fig. S7. Production of reactive nitrogen species (RNS) in conditioned medium (CM) of HEK cells transfected with iNOS.** HEK 293 cells were co-transfected with iNOS plus either WT C3 or non-nitrosylatable mutant C3(C720A) in the presence and absence of 1 mM L-NAME. The CM from these cells was collected, and nitrite/nitrate levels were determined using Griess reagent. Values are mean ± SEM. Statistical significance evaluated by ANOVA with post hoc Tukey's test. ***$P < 0.001$; ns: not significant, $n = 3$ independent experiments.

**Table S1. Demographic characteristics of human brains from AD patients and non-AD controls in the current study.**

| PATH_ID | Clinic_DX | Braak1 | Age | Sex | PM HRS | ApoE | Education | PATH_DX | Source |
|---|---|---|---|---|---|---|---|---|---|
| 5548 | AD | 6 | 80 | M | 6 | --- | 16 | Alzheimers disease | UCSD Medical Center |
| 5443 | AD | 6 | 84 | M | 8 | --- | 11 | Alzheimers disease | UCSD Medical Center |
| 5394 | AD | 6 | 81 | M | 4 | --- | 20 | Alzheimers disease | UCSD Medical Center |
| 5680 | AD | 6 | 84 | M | 12 | 44 | --- | Alzheimers changes | UCSD Medical Center |
| 5626 | AD | 6 | 83 | M | 3 | 34 | 18 | Alzheimers changes | UCSD Medical Center |
| 5594 | AD | 6 | 83 | M | 5 | 33 | 12 | Alzheimers disease | UCSD Medical Center |
| 5342 | AD | 6 | 83 | M | 10 | 33 | 20 | Alzheimers disease | UCSD Medical Center |
| 5268 | AD | 6 | 84 | M | 6 | 34 | 12 | Alzheimers disease | UCSD Medical Center |
| 5321 | AD | 6 | 84 | M | --- | 33 | 13 | Alzheimers disease | UCSD Medical Center |
| 5577 | AD | 5 | 83 | M | 10 | 44 | 15 | Alzheimers disease | UCSD Medical Center |
| 5713 | Non-AD | 1 | 81 | M | 12 | --- | 16 | Frontotemporal Lobar Degeneration-TDP43 | UCSD Medical Center |
| 5687 | Non-AD | 1 | 84 | M | 8 | --- | 14 | Normal | UCSD Medical Center |
| 5529 | Non-AD | 1 | 81 | M | 12 | 33 | 16 | Alzheimers changes | UCSD Medical Center |
| 5462 | Non-AD | 2 | 81 | M | 8 | --- | 16 | Dementia with Lewy bodies | UCSD Medical Center |
| 5452 | Non-AD | 1 | 83 | M | 12 | --- | 13 | Alzheimers changes | UCSD Medical Center |
| 5329 | Non-AD | 2 | 84 | M | 12 | --- | 12 | Lewy body variant of Alzheimers disease | UCSD Medical Center |
| 4982 | Non-AD | 1 | 81 | M | 10 | --- | 18 | Alzheimers changes | UCSD Medical Center |
| 4942 | Non-AD | 0 | 83 | M | --- | 33 | 16 | Normal | UCSD Medical Center |
| 5783 | Non-AD | 2 | 84 | M | 36 | 23 | 16 | Normal | UCSD Medical Center |
| 5130 | Non-AD | --- | 71 | M | 2 | -4 | -4 | Normal | UCSD Medical Center |
| 5764 | AD | 6 | 83 | F | 8 | --- | 20 | Alzheimers disease | UCSD Medical Center |
| 5738 | AD | 6 | 85 | F | 6 | --- | 16 | Alzheimers disease | UCSD Medical Center |
| 5731 | AD | 6 | 84 | F | 8 | --- | 15 | Alzheimers disease | UCSD Medical Center |
| 5657 | AD | 6 | 85 | F | 6 | --- | 18 | Alzheimers disease | UCSD Medical Center |
| 5636 | AD | 6 | 81 | F | 8 | --- | 16 | Alzheimers disease | UCSD Medical Center |
| 5566 | AD | 6 | 84 | F | 6 | 33 | 12 | Alzheimers disease | UCSD Medical Center |
| 5303 | AD | 6 | 83 | F | 5 | --- | 14 | Alzheimers disease | UCSD Medical Center |
| 5269 | AD | 6 | 83 | F | 12 | --- | 13 | Alzheimers disease | UCSD Medical Center |
| 5252 | AD | 6 | 83 | F | 6 | --- | 16 | Alzheimers disease | UCSD Medical Center |
| 5238 | AD | 6 | 82 | F | 8 | --- | -4 | Alzheimers disease | UCSD Medical Center |
| 4734 | Non-AD | 1 | 79 | F | 2 | 33 | -4 | Lewy body variant of Alzheimers disease | UCSD Medical Center |
| 4806 | Non-AD | 1 | 76 | F | 15 | 33 | 14 | Diffuse Lewy body disease | VA Medical Center- San Diego |
| 4876 | Non-AD | 1 | 82 | F | --- | 33 | 7 | Degeneration of substantia nigra | VA Medical Center- San Diego |
| 4904 | Non-AD | 1 | 78 | F | --- | 23 | 9 | Fronto-temporal atrophy without Pick bodies | VA Medical Center- San Diego |
| 4980 | Non-AD | 2 | 79 | F | --- | 33 | 12 | Fronto-temporal atrophy without Pick bodies | UCSD Medical Center |
| 5014 | Non-AD | 2 | 82 | F | 9 | 33 | 19 | Progressive supranuclear palsy | UCSD Medical Center |
| 5074 | Non-AD | 2 | 85 | F | --- | --- | 12 | Lewy body variant of Alzheimers disease | UCSD Medical Center |
| 5080 | Non-AD | 1 | 84 | F | 9 | 24 | 17 | Not demented | UCSD Medical Center |
| 5341 | Non-AD | 0 | 77 | F | 12 | --- | 12 | Normal | UCSD Medical Center |
| 5827 | Non-AD | 0 | 80 | F | 7 | --- | 20 | Frontotemporal Lobar Degeneration - tau | UCSD Medical Center |

**Table S2. Demographics of all human brain donors.**

| Category | Non-AD | AD | *P*-value |
|---|---|---|---|
| **Age** | **80.75 ± 3.4** | **83.1 ± 1.3** | **0.0064** |
| **Sex (M/F)** | **10/10** | **10/10** | **0.9999** |
| **PMI** | **11.07 ± 7.8** | **7.21 ± 2.1** | **0.0501** |

Mean ± SD; M, male; F, female; a t-test was used to detect differences in age and PMI, while a $\chi^2$ was used for gender difference between the groups. While the mean age between the AD and non-AD control groups was statistically different, this may not be biologically meaningful among octogenarians.

**Table S3. SNO-proteins identified in the brains of each of the 4 experimental groups.** These groups were comprised of autopsy-confirmed male and female AD patients, and corresponding non-AD controls matched for age, sex, education, and ethnicity. See separate, appended EXCEL file.

**Table S4. Lists of SNO-proteins found in male AD, female AD, and their respective non-AD control brains.** Data include identified peptides, SNO modification sites and distribution among brains from AD and non-AD individuals. Note that the category "non-specific SNO-proteins" refers to false-positive proteins detected by mass spectrometry in the absence of *SNO*TRAP reagent in 4 technical replicates. See separate, appended EXCEL file.

**Table S5. Complete lists of SNO-proteins identified in the brain of each individual in the study.** See separate, appended EXCEL file.

**Table S6. Lists of SNO-proteins found exclusively in male AD or in female AD brains.** See separate, appended EXCEL file.

**Table S7. SNO-proteins identified with significantly-different abundance between AD patients and non-AD controls, including false-discovery rates, *P*-values, and global summary.** See separate, appended EXCEL file.

**Table S8. Rank list of significantly upregulated SNO-proteins in all AD brains compared to non-AD brains.** See separate, appended EXCEL file.

**Table S9. List of significantly increased SNO-proteins in normal vs. AD brains.** See separate, appended EXCEL file.

**Table S10. Complete list of SNO-proteins identified in AD brains.** FAD, female AD brains; MAD, male AD brains. "Exclusive" indicates present in one sex and not the other. See separate, appended EXCEL file.

**Table S11. Summary of GO processes and pathway analyses for SNO-proteins found in AD patient brains and non-AD controls.** FC, control female non-AD brains; MC, control male non-AD brains; FAD, female AD brains; MAD, male AD brains. See separate, appended EXCEL file.

**Table S12. Network clusters for female and male AD brains and respective controls.** RCTM, enrichment reactomes; FC, control female non-AD brains; MC, control male non-AD brains; FAD, female AD brains; MAD, male AD brains. See separate, appended EXCEL file.

**Table S13. Summary of GO processes and pathway analyses for SNO-proteins found exclusively in female AD brains or exclusively in male AD brains (compared to each other),**

**and in common between these brains.** FAD, female AD brains; MAD, male AD brains. See separate, appended EXCEL file.

**Table S14. Network cluster analyses for SNO-proteins found exclusively in female AD brain or exclusively in male AD brains, or in common between these brains.** RCTM, enrichment reactomes; FAD, female AD brains; MAD, male AD brains. See separate, appended EXCEL file.

**Table S15. Accuracy of peptide fragments showing S-nitrosylation in parts per million (ppm).**

Cys44 for p62, Cys720 for C3, Cys487 for PLD3, and Cys1018 for NRXN3.

### p62

| Fragment ions | Theoretical *m/z* | Observed *m/z* | Δppm |
|---|---|---|---|
| PGPC*ER ($y_6$) | 783.3458 | 783.3447 | -1.4 |
| GPGPC*ER ($y_7$) | 840.3673 | 840.3656 | -0.8 |
| AGPGPC*ER ($y_8$) | 911.4044 | 911.4010 | -3.7 |
| AAGPGPC*ER ($y_9$) | 982.4415 | 982.4341 | -7.5 |
| FSFCCSPE ($b_8$) | 1015.3653 | 1015.3704 | 5.0 |
| AAAGPGPC*ER ($y_{10}$) | 1053.4786 | 1053.4801 | 1.4 |

### C3

| Fragment ions | Theoretical *m/z* | Observed *m/z* | Δ ppm |
|---|---|---|---|
| K ($y_1$) | 147.1133 | 147.1129 | -2.7 |
| FI ($b_2$) | 261.1603 | 261.1601 | -0.8 |
| C*K ($y_2$) | 375.1702 | 375.1698 | -1.0 |
| AC*K ($y_3$) | 446.2073 | 446.2076 | 0.7 |
| GEAC*K ($y_5$) | 632.2713 | 632.2714 | 0.2 |
| SLGEAC*K ($y_7$) | 832.3874 | 832.3870 | -0.5 |

### PLD3

| Fragment ions | Theoretical *m/z* | Observed *m/z* | Δ ppm |
|---|---|---|---|
| R ($y_1$) | 175.1195 | 175.1194 | -0.6 |
| DW ($a_2$) | 274.1191 | 274.1199 | 2.9 |
| DW ($b_2$) | 302.1140 | 302.1137 | -1.0 |
| DWD ($b_3$) | 417.1410 | 417.1410 | 0.0 |
| NAC*R ($y_4$) | 588.2563 | 588.2596 | 5.6 |
| GNAC*R ($y_5$) | 645.2778 | 645.2786 | 1.2 |
| VGNAC*R ($y_6$) | 744.3461 | 744.3481 | 2.7 |
| PYSHDLD | 828.3527 | 828.3538 | 1.3 |
| SPYSHDLD | 915.3847 | 915.3830 | -1.9 |
| DSVGNAC*R ($y_8$) | 946.4051 | 946.4064 | 1.4 |
| ADSVGNAC*R ($y_9$) | 1017.4422 | 1017.4431 | 0.9 |
| DSPYSHDLD | 1030.4116 | 1030.4105 | -1.1 |
| DWDSPYSHD ($b_9$) | 1103.4069 | 1103.4063 | -0.5 |
| TSADSVGNAC*R ($y_{11}$) | 1205.5220 | 1205.5112 | -9.0 |
| DTSADSVGNAC*R ($y_{12}$) | 1320.5488 | 1320.5419 | -5.2 |
| DWDSPYSHDLD ($b_{11}$) | 1331.5178 | 1331.5144 | -2.6 |
| DLDTSADSVGNAC*R ($y_{12}$) | 1548.6598 | 1548.6699 | 6.5 |

Asterisks indicate modification sites.

### NRXN3

| Fragment ions | Theoretical *m/z* | Observed *m/z* | Δ ppm |
|---|---|---|---|
| R ($y_1$) | 175.1195 | 175.1196 | 0.6 |
| FQ | 276.1348 | 276.1356 | 2.9 |
| DGF ($b_3$) | 320.1246 | 320.1254 | 2.5 |
| NGR ($y_3$) | 346.1838 | 346.1848 | 2.9 |
| LNGR ($y_4$) | 459.2679 | 459.2674 | -1.1 |
| DLNGR ($y_5$) | 574.2948 | 574.2957 | 1.6 |
| VDLNGR ($y_6$) | 673.3632 | 673.3680 | 7.1 |
| SVDLNGR ($y_7$) | 760.3952 | 760.3949 | -0.4 |
| ASVDLNGR ($y_8$) | 831.4324 | 831.4214 | 0 |
| LASVDLNGR ($y_9$) | 944.5164 | 944.5190 | 2.8 |
| C*LASVDLNGR ($y_{10}$) | 1172.5732 | 1172.5717 | -1.3 |
| GC*LASVDLNGR ($y_{11}$) | 1229.5946 | 1229.5977 | 2.5 |
| QGC*LASVDLNGR ($y_{12}$) | 1357.6532 | 1357.6541 | 0.7 |
